# Hepatitis B prevalence, knowledge and attitudes among health-care workers and antenatal mothers attending a tertiary hospital in South Tarawa, Kiribati: insights from a 2022 cross-sectional study

**DOI:** 10.5365/wpsar.2025.16.4.1242

**Published:** 2025-12-18

**Authors:** Thomas Russell, Vikash Sharma, Alice Lee

**Affiliations:** a Department of Internal Medicine, Tungaru Central Hospital, Ministry of Health and Medical Services, Tarawa, Kiribati.; b School of Medical Sciences, College of Medicine, Nursing and Health Sciences, Fiji National University, Suva, Fiji.; c Department of Gastroenterology and Liver Services, Concord Repatriation General Hospital, University of Sydney, Sydney, New South Wales, Australia.

## Abstract

**Objective:**

Hepatitis B virus infection is hyperendemic in Kiribati (~15% prevalence rate), with vaccination and antiviral treatment being the mainstays of control. Prevalence, knowledge and attitudes among health-care workers and antenatal mothers are poorly understood.

**Methods:**

A cross-sectional, descriptive study was conducted among health-care workers and antenatal mothers at Tungaru Central Hospital on South Tarawa, Kiribati in 2022. The study included hepatitis B virus serology and a bilingual questionnaire.

**Results:**

Fifty-one health-care workers and 49 women receiving antenatal care participated in the study. Most health-care workers (98.0%) had heard of the hepatitis B virus and most (54.9%) exhibited a moderate level of knowledge. Less than half (46.9%) of the antenatal mothers had heard of the hepatitis B virus and most (63.3%) had a low level of knowledge. Most health-care workers (60.8%) and half of antenatal mothers (49.0%) had satisfactory attitudes towards screening, care-seeking and vaccination, and 93.9% approved of adult catch-up vaccination. Hepatitis B virus prevalence was 23.0% (15.7% of health-care workers, 30.6% of antenatal mothers).

**Discussion:**

Extensive educational campaigns for antenatal mothers are needed to enhance awareness of the infection, while training for health-care workers on transmission, prevention and treatment is critical for informing and galvanizing action on hepatitis B virus.

Hepatitis B virus (HBV) was declared a global public health threat in 2016 by the World Health Organization (WHO). (*1*, *2*) Global elimination goals have been set with targets to be achieved by 2030. (*1*) HBV is an enveloped virus that primarily infects the liver and can cause both acute and chronic infection. (*3*, *4*) Its transmission is via blood and body fluids, with mother-to-child transmission (MTCT) during delivery being the most common route in developing countries. (*4*) Other transmission routes are sexual encounters with an infected partner, needle-stick injuries and unsafe infection control practices. (*4*) WHO estimates that, in 2022, approximately 304 million people lived with chronic HBV infection, with 1.2 million new infections annually and over 1 million deaths attributable to cirrhosis and hepatocellular carcinoma. (*4*) Treatment is highly effective, with long-term therapy required in the majority of cases. (*5*) Functional cure is seen in some patients, in which cessation of antiviral treatment is possible. (*5*) Hence, preventive strategies remain central in achieving elimination with universal provision of timely infant HBV birth-dose vaccination, preferably within 24 hours of birth, followed by three subsequent doses at least 4 weeks apart. (*4*)

The WHO Western Pacific Region is home to a quarter of the world’s population and bears the highest burden of HBV globally, with a prevalence rate of 7%. (*2*, *6*) Pacific island countries and territories (PICTs) have disproportionately higher prevalence rates compared to the more developed counterparts within the Region. (*7*-*10*) The majority of deaths in the Region caused by viral hepatitis are attributed to HBV, including deaths from acute hepatitis (72%), liver cancer (53%) and liver cirrhosis (30%), according to 2019 WHO data. (*7*) Although inroads have been made in addressing the burden of disease among PICTs with several inaugural treatment programmes having been established, access to treatment remains challenging. (*6*, *11*, *12*) There are limited studies published on HBV in PICTs.

Kiribati is a large ocean state PICT comprised of 33 coral atolls scattered over an expanse of 3.5 million square kilometres in the Pacific Ocean, making it one of the most remote locations in the world – both internationally and domestically. (*13*, *14*) The country has a population of over 135 000 and provides free health care. (*15*) Its vulnerability to the effects of climate change is well documented, and it is one of three PICTs designated as a least developed country by the United Nations. (*16*, *17*) Hyperendemic in Kiribati, HBV has a pooled prevalence rate of 15%, (*10*, *18*) doubling regional averages, and compounded by high rates of hepatitis D virus (HDV) coinfection (~41%). (*10*) A hepatitis B treatment programme in the country has existed since 2018, which supports screening efforts and antiviral treatment using contextualized WHO guidelines that include antenatal mothers (ANMs), and prioritizes health-care worker (HCW) screening, vaccination and access to antiviral therapy, as appropriate. (*11*)

The Western Pacific Region and its Member States, which includes Kiribati, have followed the WHO Expanded Programme on Immunization (EPI) with the gradual and decisive introduction of vaccination against vaccine-preventable diseases (VPDs) to PICTs, as outlined in the *Regional strategic framework for vaccine-preventable diseases and immunization in the Western Pacific 2021–2030*. (*19*) Universal vaccination of newborns was introduced in 1989 to Kiribati; however, supplies were often insufficient or inconsistently available. (*20*) By 1995, a project managed by the United Nations International Children’s Fund (UNICEF) and supported by WHO helped to establish a reliable supply chain and technical support for 10 PICTs. (*18*) To date, the immunization schedule specifies vaccinations at birth and at 6, 10 and 14 weeks of age. The hepatitis B vaccine administered at birth is a monovalent vaccine, while a pentavalent (Diphtheria–tetanus–pertussis-Hib-HepB) vaccine is used for the subsequent three doses. (*21*, *22*)

The ANMs and HCWs form an important population cohort pivotal to addressing ongoing transmission of HBV. Transmission of HBV at birth via MTCT is the most common route, with preventive measures in place to mitigate its impact, while the nature of a HCW’s occupation exposes them to increased risks of contracting as well as transmitting the virus. Existing national guidelines and policies reflect the importance of HBV screening among ANMs and HCWs. (*23*, *24*) The latest local data on HBV prevalence of 9% is from a WHO 2002 serosurvey of ANMs that was part of a sexual transmission infection study. (*25*) To date, there are no official data on the prevalence of HBV among HCWs in Kiribati. Prevention of HBV remains the mainstay of control efforts, and the knowledge and attitudes of HCWs and ANMs are key. Given the lack of existing data on this topic, the objectives of this study were to assess the prevalence, knowledge and attitudes towards HBV among HCWs working on the capital island of South Tarawa and ANMs attending an antenatal clinic at the tertiary-care national referral hospital, Tungaru Central Hospital (TCH).

## Methods

### Study design, setting and sample

A cross-sectional study was conducted over a 3-month period from 1 September to 30 November 2022. The study site was TCH located in Nawerewere village on South Tarawa island. A convenience sampling method was employed to recruit as many participants as possible.

### Study participants

Participants invited to take part in the study were HCWs aged ≥ 18 years employed by the Ministry of Health and Medical Services (MHMS) working on South Tarawa and ANMs aged ≥ 18 years attending the antenatal clinic at TCH. All participants were required to provide written informed consent before their inclusion in the study. This was facilitated with the provision of a bilingual Participation Information Form, as well as a Withdrawal Form to emphasize the enrolled person’s voluntary participation and withdrawal at any point.

### HBV serology testing

The purpose of the study was explained to consenting participants. Approximately 2 mL of serum were drawn from the antecubital fossa of each participant and placed in a red cap (dry) tube by laboratory staff at TCH national laboratory. Samples were then tested for the presence of hepatitis B surface antigen (HBsAg) using the pre-qualified Determine^TM^ HBsAg2 immunochromatographic point-of-care (POC) test strips – sensitivity 100%, (*26*) specificity 99.6–100% (*26*, *27*) – according to the manufacturer’s instructions. Due to the limited number of POC test strips available during the study period, testing was limited to participants who either had never been tested for HBsAg or whose previous HBsAg test was older than 6 months. Documented HBsAg results that were within 6 months old and confirmed on either their antenatal folders, patient folders or TCH’s laboratory register were considered valid. Both cohorts were tested using the Determine HBsAg2 POC test strips.

### Study survey

Consenting respondents completed a structured survey adapted from Abeje and Azage (2015) (*28*) in the presence of a researcher who could answer any clarifying questions, either face-to-face or via teleconference. The survey collected data on basic sociodemographic characteristics, as well as knowledge of and attitudes towards HBV and vaccination. The survey, structured in English, was translated into Gilbertese, and was reviewed via discipline specialists to ensure relevance, readability, clarity and comprehensiveness of the knowledge and attitude items. It was subsequently piloted by 10 HCWs and 10 ANMs to assess clarity and usability and consequently modified and finalized.

### Scoring knowledge and attitude

The survey contained categorical self-assessment questions (3 levels: yes, no, not sure/I don’t know). The “not sure/I don’t know” response was used to minimize the guessing effect and was scored as incorrect. Questions covered demographic characteristics, knowledge of HBV status, risks of exposure to HBV, knowledge of HBV infection, knowledge of HBV prevention and control measures, and knowledge of HBV vaccination. There were 14 knowledge-based questions and six attitude-based questions. The total knowledge (range 0–14) and attitude (range 0–6) scores for each respondent were calculated, with 1 point given for a correct response and none for an incorrect one. Data were collected via a hybrid model – either remotely via teleconference calls or in-person at the hepatitis or antenatal clinic at TCH. The mean total knowledge and attitude scores were determined to assess the level of knowledge and attitude based on Bloom’s cutoff categories, (*29*, *30*) which are divided into three levels based on percentage scores: high-level (80–100%), moderate-level (60–79%) and low-level (< 60%). Assessment of attitude was also scored accordingly and categorized as either satisfactory (80–100%), neutral (60–79%) or unsatisfactory (< 60%). (*29*, *30*)

### Data management and analysis

Raw data were deidentified and entered into Microsoft Excel for cleaning and coding, with regular backup files saved and stored in Microsoft’s OneDrive file hosting service, which is a personalized, password-protected, secure data storage account accessible only to the primary investigator. Incomplete or missing data were omitted from the final analysis. Descriptive analyses were undertaken, with the mean and standard deviation used to summarize continuous variables, and categorical variables were summarized using frequencies and proportions.

## Results

### Sociodemographic characteristics of participants

The sociodemographic characteristics of participants are displayed in **Table 1**. A total of 103 individuals consented to participate in the study (51 HCWs, 52 ANMs), with three excluded due to incomplete data entry (1) and being underage (2). The final number for analysis was 100 (51 HCWs, 49 ANMs). Among HCWs, a plurality were in the 18–29-year age category (47.1%), and most were married (54.9%) and had achieved tertiary education (90.2%). Among ANMs, most were from the same age category as HCWs (57.1%), married (67.3%), unemployed (57.2%) and educated to the secondary level (75.5%).

**Table 1:** Demographic characteristics and HBV seroprevalence of surveyed health-care workers and antenatal mothers, South Tawara, Kiribati, 2022

Characteristic	Health-care workers (*n* = 51)		Antenatal mothers (*n* = 49)	
	*n*	%	*n*	%
Sex Female Male	32 19	62.7 37.3	49 –	100 –
Age group (years) 18–29 30–39 40–49 ≥ 50	24 21 4 2	47.1 41.2 7.8 3.9	28 19 2 –	57.1 38.8 4.1 –
Partnership status Married De facto Single Divorced Widowed	28 8 12 2 1	54.9 15.7 23.5 3.9 2.0	33 14 1 1 –	67.3 28.6 2 2 –
Education achieved Primary Secondary Tertiary Vocational	– 4 46 1	– 7.8 90.2 2.0	1 37 9 2	2.0 75.5 18.4 4.1
Employment Unemployed Nurse Medical officer (doctor) Laboratory technician Health officer Physiotherapist Government employee Self-employed Other^a^	– 26 15 8 2 – – – –	– 51.0 29.4 15.7 3.9 – – – –	28 1 – – – 1 5 2 12	57.2 2.0 – – – 2.0 10.2 4.1 24.5
HBV seroprevalence HBsAg-positive HBsAg-negative	8 43	15.7 84.3	15 34	30.6 69.4

HBsAg: hepatitis B surface antigen; HBV: hepatitis B virus.

^a^ Employed by nongovernmental entities, private companies or faith-based organizations.

### Prevalence of HBV among participants

The prevalence of HBsAg positivity among all participants was 23.0% (23/100). The HBsAg seropositivity rate was 15.7% (8/51) among HCWs. All positive cases were new cases not previously identified. The HBsAg seropositivity rate among ANMs was 30.6% (15/49). Among seropositive ANMs, 13.3% (2/15) were new cases not previously identified. **Table 1** shows the HBsAg prevalence for each cohort.

### Assessment of HBV knowledge among participants

**Fig. 1:**
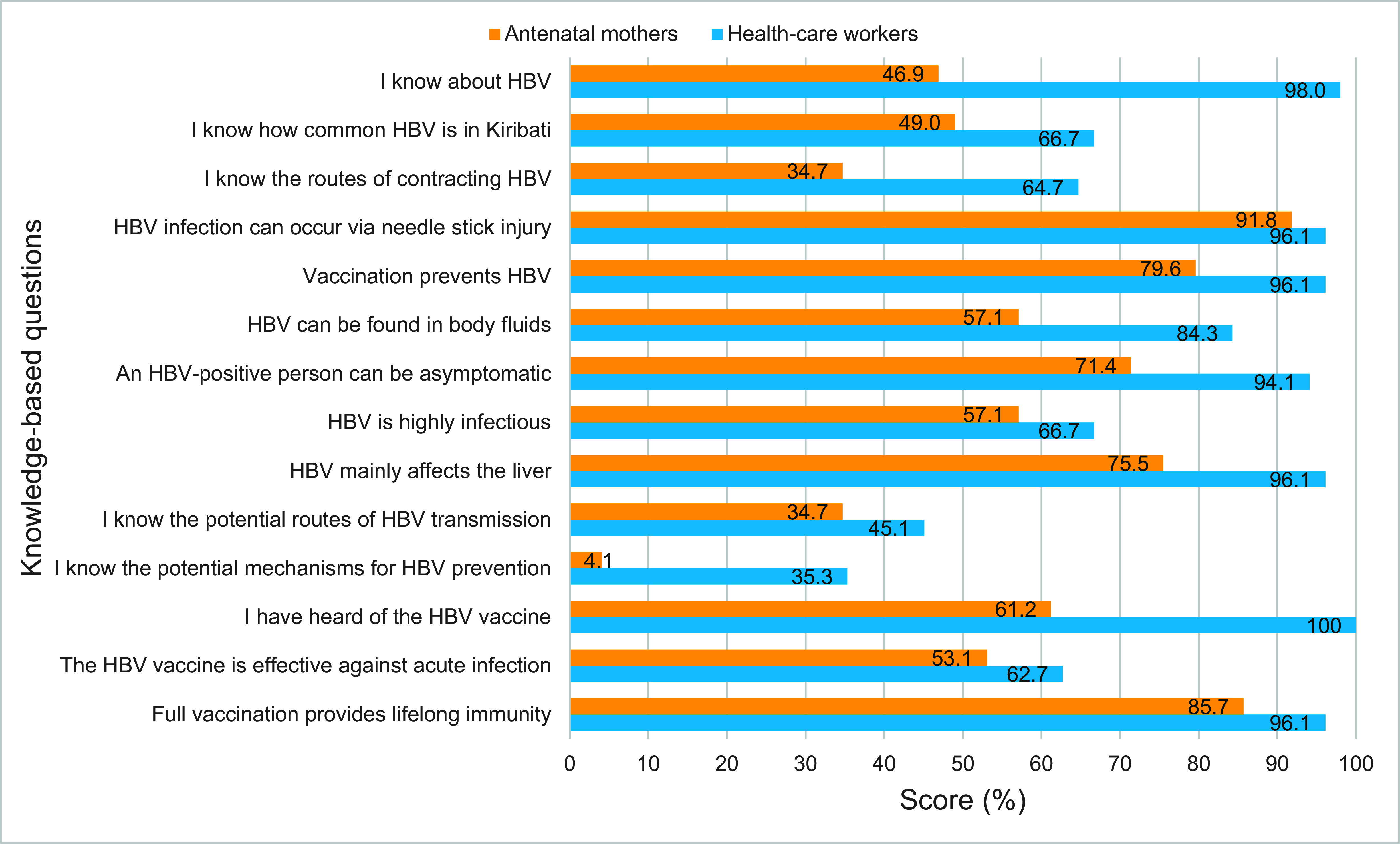
Knowledge-based scores about HBV among health-care workers (^N^ = 51) and antenatal mothers (^N^ = 49), South Tawara, Kiribati, 2022

A total of 14 questions were used to assess participants’ knowledge of HBV infection, transmission, prevention and vaccination (**Fig. 1**). Assessment of each cohort’s knowledge is presented separately below.

[**insert** Figure 1]

#### HBV knowledge among health-care workers

The mean knowledge score for HCWs was 11.0 ± 1.6 out of 14 (78.6%), with 37.3% displaying high-level knowledge and 54.9% showing moderate-level knowledge (**Table 2**). The majority of HCWs (98.0%) had heard about HBV. HCWs exhibited moderate-level knowledge of HBV prevalence in Kiribati (66.7%), infectivity of HBV (66.7%) and effectiveness of vaccination for acute infection (62.7%). HCWs had low-level knowledge when asked to correctly identify potential routes of transmission (45.1%) and mechanisms to prevent HBV (35.3%) (**Fig. 1**).

**Table 2:** HBV knowledge and attitude scores of surveyed health-care workers and antenatal mothers, South Tawara, Kiribati, 2022

Variable	Category (%)	Score	Health-care workers (*n* = 51)			Antenatal mothers (*n* = 49)		
			*n*	%	Mean ± SD	*n*	%	Mean ± SD
**Knowledge**	**High** **(80–100)**	**12**–**14**	**19**	**37.3**	**11.0 ± 1.6**	**1**	**2.0**	**7.9 ± 1.8**
	**Moderate** **(60–79)**	**9**–**11**	**28**	**54.9**		**17**	**34.7**	
	**Low** **(< 60)**	** < 9**	**4**	**7.8**		**31**	**63.3**	
**Attitude**	**Satisfactory** **(80–100)**	**5**–**6**	**31**	**60.8**	**4.7 ± 0.8**	**24**	**49.0**	**4.0 ± 0.8**
	**Neutral** **(60–79)**	**4**	**17**	**33.3**		**18**	**36.7**	
	**Unsatisfactory** **(< 60)**	** < 4**	**3**	**5.9**		**7**	**14.3**	

HBV: hepatitis B virus; SD: standard deviation.

#### HBV knowledge among antenatal mothers

The mean knowledge score for ANMs was 7.9 ± 1.8 out of 14 (56.4%), with most ANMs (63.3%) showing low-level knowledge (**Table 2**). Less than half of ANMs (46.9%) knew about HBV. ANMs exhibited moderate-level knowledge about HBV vaccination (61.2%), infectivity of HBV (57.1%) and that HBV mainly affects the liver (75.5%). ANMs had low-level knowledge concerning routes of HBV transmission (34.7%) and mechanisms to prevent HBV (4.1%) (**Fig. 1**).

### Assessment of attitudes of participants towards HBV

**Fig. 2:**
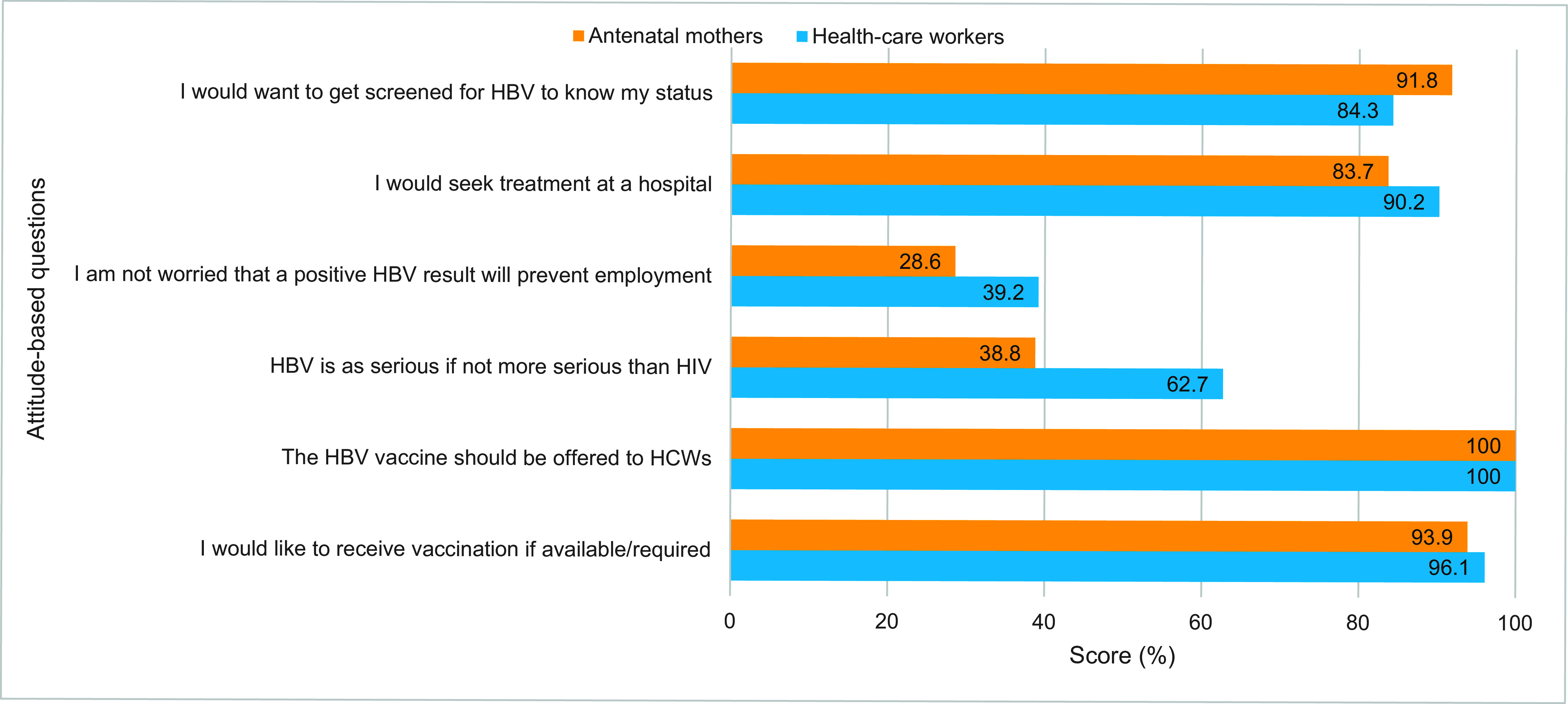
Attitude-based scores about HBV among health-care workers (^N^ = 51) and antenatal mothers (^N^ = 49), South Tawara, Kiribati, 2022

A total of six questions were posed to assess the participants’ attitudes towards HBV screening, health-seeking behaviour, severity of condition relative to human immunodeficiency virus (HIV) and vaccination (**Fig. 2**). Assessment of each cohort’s attitude is presented separately below.

[**insert** Figure 2]

#### Attitudes of health-care workers towards HBV

The mean attitude score for HCWs was 4.7 ± 0.8 out of 6 (78.3%), with the majority (60.8%) expressing satisfactory attitudes towards HBV (**Table 2**). They had satisfactory attitudes regarding HBV screening (84.3%), health-seeking behaviour (90.2%) and vaccination (96.1%) but neutral attitudes when viewing HBV as a serious condition relative to HIV (62.7%) (**Fig. 2**).

#### Attitudes of antenatal mothers towards HBV

The mean attitude score for ANMs was 4.0 ± 0.8 out of 6 (66.7%), with almost half of ANMs (49.0%) exhibiting satisfactory attitudes towards HBV (**Table 2**). They had satisfactory attitudes towards HBV screening (91.8%), health-seeking behaviour (83.7%) and vaccination (93.9%), but unsatisfactory attitudes when viewing HBV as a serious condition relative to HIV (38.8%) (**Fig. 2**).

## Discussion

The objective of this study was to assess the prevalence of HBV, as well as knowledge and attitudes towards HBV among HCWs and ANMs in a resource-limited country where HBV is hyperendemic. A high prevalence of HBV was found in both cohorts. Additionally, an underwhelming level of knowledge of HBV among HCWs and ANMs was observed, while both cohorts had satisfactory attitudes towards HBV.

The prevalence of HBsAg seropositivity was high in this study. The prevalence rate among HCWs is analogous to national rates. Among ANMs, the prevalence rate is much higher and is likely influenced by the participation of known HBsAg-positive ANMs already attending the hepatitis clinic. The small sample size may have been biased, as HBsAg-positive ANMs on South Tarawa are referred to the hospital’s antenatal clinic for care. To the best of our knowledge, our study is the first to assess knowledge of and attitudes towards HBV in a PICT and the first to provide the prevalence rate of HBV among HCWs. This prevalence rate was still higher when compared to other countries’ reported seroprevalence rates such as Cameroon (11%), (*3*) Ghana (12%), (*31*) Sudan (12%), (*32*) Ethiopia (14%), (*33*) the Solomon Islands (14%) (*34*) and Viet Nam (15–20%). (*35*) The study in Cameroon carried out HBsAg testing by using the Monalisa HBsAg ULTRA ELISA kit, (*3*) whereas the others did not specify and reported on existing national rates. Kiribati’s sustained high prevalence rates call for the MHMS to strengthen prevention of MTCT (PMTCT) strategies by ensuring HBV testing is accessible at primary health-care levels and that standardized pathways to link cases to care are followed.

Most of the HCWs exhibited a moderate level of knowledge, comparable to other studies assessing HCWs’ knowledge of HBV. In Cameroon, Akazong et al. (*3*) reported that 85% of HCWs had heard of HBV and 68% were assessed to have adequate knowledge on the route of transmission. In Ghana, Botchway et al. (*31*) found that 61% of HCWs had adequate knowledge of HBV vaccination, while in Afghanistan, Roien et al. (*36*) found that the overall knowledge score among HCWs was 87%, although insights into possible reasons for this were not highlighted. Lower scores were described by Hang Pham et al., (*35*) who noted a median knowledge score of 60% among HCWs in Viet Nam. Our research on the HCW population did not calculate a sample size, as this was the first study of its kind in the setting and such an estimate proved difficult. However, compared to the samples in the Cameroon and Ghana studies, the level of knowledge displayed was lower than expected. Despite HCWs having heard of HBV and acknowledging how common it is in the country, they exhibited limited additional knowledge. This reflects the need for ongoing awareness, investment and evaluation of HCW training on HBV. It is likely that the lack of focused health awareness and educational activities designed for HCWs contributes to this subpar level of knowledge and should be addressed.

The majority of ANMs showed a low level of knowledge, which has also been described in similar studies with larger sample sizes than ours. In Ghana, a study by Dun-Dery et al. (*37*) assessing expectant mothers’ knowledge about HBV MTCT stated that the general knowledge score was 46% and identified that young age (< 35 years), marital status, educational level, employment status and multigravidity were strong predictors of knowledge among the 450 ANMs surveyed. In Saudia Arabia, Al-Essa et al. (*38*) recruited 422 ANMs via a systematic sampling method and reported that only 47% of participants recognized blood as a transmission route when assessing pregnant women’s perceptions and attitudes towards HBV. Furthermore, two separate studies in Ethiopia, with significantly larger sample sizes determined by fixed calculations, reported that 90% (*33*) and 73% (*39*) of ANMs had poor knowledge of HBV. Given the impact of hepatitis B in Kiribati as one of the country’s most major health-care burdens, the low level of knowledge in our study was unexpected. It likely reflects the gap in awareness – among the general population as well as in hepatitis and antenatal clinics. There has also been inertia due to a lack of access to therapy until recently, with a subdued sense of urgency towards the seriousness of HBV infection. ANMs are a critical target population of PMTCT strategies. Limited knowledge will likely pose barriers towards antiviral treatment of pregnant mothers, leading to lower rates of acceptance and the risk of further transmission.

The majority of HCWs in this study exhibited satisfactory attitudes towards testing, care-seeking and vaccination – the latter being important given the availability of a HCW catch-up vaccination programme. A study conducted in Sudan (*32*) found that most participants (86%) had a favourable attitude towards HBV prevention measures. In the Cameroon study, only 44% of HCWs had a positive attitude towards HBV, possibly attributable to an inadequate level of knowledge on the route of transmission among the study population. (*3*) As the treatment programme rolls out in Kiribati, this study will help identify areas that require ongoing attention. HCWs must be supported with ongoing training, ensuring that it is undertaken in a format that is locally appropriate given the various backgrounds and training histories of the health-care workforce. Decentralized care is core to the hepatitis care effort; success is dependent on a trained health force. A multifaceted training programme including face-to-face, remote online, case-based and didactic talks is being rolled out. Ongoing capacity-building is needed, with close monitoring to increase impact.

Most of the ANMs in our study had satisfactory attitudes. This finding is comparable to the study by Gebrecherkos et al. (*39*) in Ethiopia, in which 86% of ANMs had never been screened for HBV, but 53% of respondents would seek further investigation and treatment if they were diagnosed with HBV – a positive attitude. Another study in Ethiopia by Dagnew et al. (*33*) found that 60% of ANMs were willing to be screened for HBV and that their favourable attitudes towards HBV were significantly associated with good monthly income, living in urban areas, achieving good education, being primigravid and having a history of HBV vaccination. In Kiribati, routine antenatal screening for HBsAg is only available for ANMs registered at health facilities on South Tarawa. There is limited access to HBsAg testing elsewhere, and MHMS should urgently address this service gap. Bearing in mind our study findings, perhaps satisfactory attitudes towards HBV screening and prevention reflect a strong inclination among ANMs to support mechanisms that are offered as beneficial and protective for their unborn child. Another reason is that it may reflect general societal acceptance of HBV and rational health recommendations that are meant to preserve life. Further research in this domain will be needed to evaluate these insights.

There were several limitations to this study. First, it covered a cross-section of HCW and ANM participants from an urban setting, where health-care services and information are relatively more accessible than in rural settings, and the results cannot be generalized. Second, the use of a convenience sampling method enabled known HBV patients to participate in the study, which influenced the overall HBsAg prevalence rate of the study. Third, the limited number of HBsAg tests during the study limited the sample size, making the reported seroprevalence rate less predictive of the entire population.

### Conclusion

This study reaffirms the previously reported high rates of HBV among ANMs (31%) and provides insights into the possible high rates among HCWs (16%). The demonstrated moderate level of knowledge among HCWs and the low level of knowledge among ANMs illustrate the need for enhanced training, awareness and education on HBV for both cohorts. It is recommended that the national hepatitis programme, supported by the MHMS, conducts training workshops addressing HBV surveillance and management for all HCWs involved in antenatal services on South Tarawa. Training needs to highlight PMTCT, which will upskill HCWs and enhance the necessary theoretical knowledge to allow them to confidently impart knowledge to ANMs Crucial to this, HBsAg POC testing capacity must be available at all health facilities in the country and be enabled by: trained and motivated staff; the establishment of reliable distribution systems; and the use of secure data recording and reporting systems. Finally, the catch-up HBV vaccination afforded to HCWs should also be provided to all eligible women of  child-bearing age, before pregnancy. Central to these activities would be sufficient funding for a sustainable programme which, for a resource-constrained setting such as Kiribati, would mean not only the engagement of willing development partners but also strong political will and commitment to the necessary budgetary allocation.

## References

[1] Combating hepatitis B and C to reach elimination by 2030: advocacy brief. Geneva: World Health Organization; 2016. Available from: https://iris.who.int/handle/10665/206453, accessed 9 September 2023.

[2] Sheena BS, Hiebert L, Han H, Ippolito H, Abbasi-Kangevari M, Abbasi-Kangevari Z, et al.; GBD 2019 Hepatitis B Collaborators. Global, regional, and national burden of hepatitis B, 1990-2019: a systematic analysis for the Global Burden of Disease Study 2019. Lancet Gastroenterol Hepatol. 2022 Sep;7(9):796–829. 10.1016/S2468-1253(22)00124-8 35738290 PMC9349325

[3] Akazong W E, Tume C, Njouom R, Ayong L, Fondoh V, Kuiate JR. Knowledge, attitude and prevalence of hepatitis B virus among healthcare workers: a cross-sectional, hospital-based study in Bamenda Health District, NWR, Cameroon. BMJ Open. 2020 Mar 18;10(3):e031075. 10.1136/bmjopen-2019-031075 32193257 PMC7150593

[4] Global hepatitis report 2024: action for access in low- and middle-income countries. Geneva: World Health Organization; 2024. Available from: https://iris.who.int/handle/10665/376461, accessed 9 May 2025.

[5] Guidelines for the prevention, care, and treatment of persons with chronic hepatitis B infection. Geneva: World Health Organization; 2015. Available from: https://iris.who.int/handle/10665/154590, accessed 9 September 2023. 26225396

[6] Regional action plan for viral hepatitis in the Western Pacific 2016–2020: a priority action plan for awareness, surveillance, prevention and treatment of viral hepatitis in the Western Pacific Region. Manila: WHO Regional Office for the Western Pacific; 2016. Available from: https://iris.who.int/handle/10665/208337, accessed 9 September 2023.

[7] Hepatitis data and statistics in the Western Pacific. Manila: WHO Regional Office for the Western Pacific; 2021. Available from: https://www.who.int/westernpacific/health-topics/hepatitis/regional-hepatitis-data, accessed 9 September 2023.

[8] Speed BR, Dimitrakakis M, Thoma K, Gust ID. Control of HBV and HDV infection in an isolated Pacific Island: 1. Pattern of infection. J Med Virol. 1989 Sep;29(1):13–9. 10.1002/jmv.1890290104 2584956

[9] Tibbs CJ. Delta hepatitis in Kiribati: a pacific focus. J Med Virol. 1989 Oct;29(2):130–2. 10.1002/jmv.1890290210 2600590

[10] Jackson K, Tekoaua R, Holgate T, Edwards R, Yuen L, Lee A, et al. Hepatitis B and D in the Pacific Islands of Kiribati. J Clin Virol. 2020 Aug;129:104527. 10.1016/j.jcv.2020.104527 32645613

[11] Lee AU, Jackson K, Tekoaua R, Lee C, Huntley MS, Hilmers DC. A programme to treat chronic hepatitis B in Kiribati: progress and challenges. West Pac Surveill Response. 2020 Sep 30;11(3):21–5. 10.5365/wpsar.2019.10.4.003 33936856 PMC8053900

[12] Lee AU, Mair L, Kevin B, Gandi L, Tarumuri O, Lee C, et al. Prevalence of chronic hepatitis B in Oro Province, Papua New Guinea. West Pac Surveill Response. 2020 Dec 16;11(4):6–9. 10.5365/wpsar.2020.11.3.001 34046236 PMC8152820

[13] Casey ST, Cook AT, Ferguson MM, Noste E, Mweeka KT, Rekenibai TE, et al. Strengthening health emergency response capacity in Kiribati: establishing the Kiribati medical assistance team (KIRIMAT). Western Pac Surveill Response J. 2023;14(6 Spec edition):1–3. doi:10.5365/wpsar.2023.14.6.1013 37197089 PMC10184053

[14] Kiribati: remote island nation faces a triple threat to health [news release]. Medecins Sans Frontieres; 2023. Available from: https://www.doctorswithoutborders.org/latest/kiribati-remote-island-nation-faces-triple-threat-health, accessed 9 September 2023.

[15] Kiribati. Noumea: The Pacific Community [Internet]; 2025. Available from: https://www.spc.int/our-members/kiribati/details, accessed 18 November 2025.

[16] Ives M. A remote Pacific nation, threatened by rising seas. The New York Times [Internet]; 2 July 2016. Available from: https://www.nytimes.com/2016/07/03/world/asia/climate-change-kiribati.html, accessed 9 September 2023.

[17] The least developed countries report 2022: the low-carbon transition and its daunting implications for structural transformation. Geneva: United Nations Conference on Trade and Development; 2022. Available from: https://unctad.org/publication/least-developed-countries-report−2022, accessed 9 September 2023.

[18] Wilson N, Ruff TA, Rana BJ, Leydon J, Locarnini S. The effectiveness of the infant hepatitis B immunisation program in Fiji, Kiribati, Tonga and Vanuatu. Vaccine. 2000 Jul 1;18(26):3059–66. 10.1016/S0264-410X(00)00080-3 10825610

[19] Regional strategic framework for vaccine-preventable diseases and immunization in the Western Pacific 2021–2030. Manila: WHO Regional Office for the Western Pacific; 2022. Available from: https://iris.who.int/handle/10665/359540, accessed 9 September 2023.

[20] Viral hepatitis situation and response in Kiribati 2015. Manila: WHO Regional Office for the Western Pacific; 2017. Available from: https://iris.wpro.who.int/handle/10665.1/13615, accessed 9 September 2023.

[21] Condon R. Options for Australia and New Zealand development assistance in health, Kiribati: concept note. Canberra: Health Resource Facility for Australia’s Aid Program; 2014. Available from: https://www.mfed.gov.ki/sites/default/files/2025−06/140214c%20Kiribati%20Health%20Concept%20Note%20final.pdf, accessed 21 August 2024.

[22] Kiribati annual health bulletin 2022. Tarawa: Ministry of Health and Medical Services; 2022. Available from: https://sdd.spc.int/digital_library/2022-kiribati-annual-health-bulletin, accessed 9 September 2023.

[23] National reproductive, maternal, newborn, child and adolescent health policy, strategy & implementation plan. Tarawa: Ministry of Health and Medical Services; 2022.

[24] Kiribati guidelines for the management of healthcare workers known to be infected with hepatitis B, hepatitis C, HIV. Tarawa: Ministry of Health and Medical Services; 2018.

[25] Prevalence surveys of sexually transmitted infections among seafarers and women attending antenatal clinics in Kiribati: 2002–2003. Manila: WHO Regional Office for the Western Pacific; 2004. Available from: https://iris.wpro.who.int/handle/10665.1/5372, accessed 9 September 2023.

[26] WHO prequalification of in vitro diagnostics: public report. Geneva: World Health Organization; 2025. Available from: https://extranet.who.int/prequal/WHOPR/public-report-determine-hbsag−2-pqdx−0451−013−00, accessed 9 May 2025.

[27] Determine™ HBsAg2 [website]. Abbott; 2025. Available from: https://www.globalpointofcare.abbott/ww/en/product-details/determine-hbsag−2.html, accessed 9 August 2025.

[28] Abeje G, Azage M. Hepatitis B vaccine knowledge and vaccination status among health care workers of Bahir Dar City Administration, Northwest Ethiopia: a cross sectional study. BMC Infect Dis. 2015 Jan 31;15(1):30. 10.1186/s12879-015-0756-8 25637342 PMC4324798

[29] Alzahrani MM, Alghamdi AA, Alghamdi SA, Alotaibi RK. Knowledge and attitude of dentists towards obstructive sleep apnea. Int Dent J. 2022 Jun;72(3):315–21. 10.1016/j.identj.2021.05.004 34193341 PMC9275360

[30] Chand D, Mohammadnezhad M, Khan S. Levels and predictors of knowledge, attitude, and practice regarding health hazards with barber’s profession in Fiji. Inquiry. 2022 Jan-Dec;59:469580221100148. 10.1177/00469580221100148 35499518 PMC9066629

[31] Botchway ET, Agyare E, Seyram L, Owusu KK, Mutocheluh M, Obiri-Yeboah D. Prevalence and attitude towards hepatitis B vaccination among healthcare workers in a tertiary hospital in Ghana. Pan Afr Med J. 2020 Aug 5;36:244. 10.11604/pamj.2020.36.244.24085 33014240 PMC7519783

[32] Mursy SMM, Mohamed SOO. Knowledge, attitude, and practice towards Hepatitis B infection among nurses and midwives in two maternity hospitals in Khartoum, Sudan. BMC Public Health. 2019 Nov 29;19(1):1597. 10.1186/s12889-019-7982-8 31783744 PMC6884767

[33] Dagnew M, Million Y, Destaw B, Adefris M, Moges F, Tiruneh M. Knowledge, attitude, and associated factors towards vertical transmission of hepatitis B virus among pregnant women attending antenatal care in tertiary hospitals in Amhara Region, Northwest Ethiopia: a cross-sectional study. Int J Womens Health. 2020 Oct 16;12:859–68. 10.2147/IJWH.S273560 33116935 PMC7585551

[34] Getahun A, Baekalia M, Panda N, Lee A, Puiahi E, Khan S, et al. Seroprevalence of hepatitis B surface antigen in pregnant women attending antenatal clinic in Honiara Solomon Islands, 2015. World J Hepatol. 2016 Dec 8;8(34):1521–8. 10.4254/wjh.v8.i34.1521 28008343 PMC5143433

[35] Hang Pham TT, Le TX, Nguyen DT, Luu CM, Truong BD, Tran PD, et al. Knowledge, attitudes and medical practice regarding hepatitis B prevention and management among healthcare workers in Northern Vietnam. PLoS One. 2019 Oct 14;14(10):e0223733. 10.1371/journal.pone.0223733 31609983 PMC6791544

[36] Roien R, Mousavi SH, Ozaki A, Baqeri SA, Hosseini SMR, Ahmad S, et al. Assessment of knowledge, attitude, and practice of health-care workers towards hepatitis B virus prevention in Kabul, Afghanistan. J Multidiscip Healthc. 2021 Nov 15;14:3177–86. 10.2147/JMDH.S334438 34815672 PMC8605488

[37] Dun-Dery F, Adokiya MN, Walana W, Yirkyio E, Ziem JB. Assessing the knowledge of expectant mothers on mother-to-child transmission of viral hepatitis B in Upper West region of Ghana. BMC Infect Dis. 2017 Jun 12;17(1):416. 10.1186/s12879-017-2490-x 28606057 PMC5469103

[38] Al-Essa M, Alyahya A, Al Mulhim A, Alyousof A, Al-Mulhim M, Essa A. Perception of and attitude towards hepatitis B infection among Saudi pregnant females attending antenatal care unit in Al-Ahsa City, Kingdom of Saudi Arabia. Cureus. 2020 Jan 16;12(1):e6673. 10.7759/cureus.6673 31976187 PMC6968831

[39] Gebrecherkos T, Girmay G, Lemma M, Negash M. Knowledge, attitude, and practice towards hepatitis B virus among pregnant women attending antenatal care at the University of Gondar Comprehensive Specialized Hospital, Northwest Ethiopia. Int J Hepatol. 2020 Jan 15;2020:5617603. 10.1155/2020/5617603 32015916 PMC6985934

